# A quantitative systems pharmacology (QSP) model for *Pneumocystis* treatment in mice

**DOI:** 10.1186/s12918-018-0603-9

**Published:** 2018-07-17

**Authors:** Guan-Sheng Liu, Richard Ballweg, Alan Ashbaugh, Yin Zhang, Joseph Facciolo, Melanie T. Cushion, Tongli Zhang

**Affiliations:** 1 Department of Pharmacology and Systems Physiology, College of Medicine, University of Cincinnati, 231 Albert Sabin Way, Cincinnati, OH 45267-0576 USA; 2 Department of Internal Medicine, College of Medicine, University of Cincinnati, Cincinnati, OH USA; 3 Division of Biostatistics and Epidemiology, Cincinnati Children’s Hospital Medical Center, Cincinnati, OH USA

**Keywords:** *Pneumocystis* - systems biology - quantitative systems pharmacology, Infectious disease

## Abstract

**Background:**

The yeast-like fungi *Pneumocystis,* resides in lung alveoli and can cause a lethal infection known as *Pneumocystis pneumonia* (PCP) in hosts with impaired immune systems. Current therapies for PCP, such as trimethoprim-sulfamethoxazole (TMP-SMX), suffer from significant treatment failures and a multitude of serious side effects. Novel therapeutic approaches (i.e. newly developed drugs or novel combinations of available drugs) are needed to treat this potentially lethal opportunistic infection. Quantitative Systems Pharmacological (QSP) models promise to aid in the development of novel therapies by integrating available pharmacokinetic (PK) and pharmacodynamic (PD) knowledge to predict the effects of new treatment regimens.

**Results:**

In this work, we constructed and independently validated PK modules of a number of drugs with available pharmacokinetic data. Characterized by simple structures and well constrained parameters, these PK modules could serve as a convenient tool to summarize and predict pharmacokinetic profiles. With the currently accepted hypotheses on the life stages of *Pneumocystis*, we also constructed a PD module to describe the proliferation, transformation, and death of *Pneumocystis*. By integrating the PK module and the PD module, the QSP model was constrained with observed levels of asci and trophic forms following treatments with multiple drugs. Furthermore, the temporal dynamics of the QSP model were validated with corresponding data.

**Conclusions:**

We developed and validated a QSP model that integrates available data and promises to facilitate the design of future therapies against PCP.

## Background

*Pneumocystis* is a common opportunistic infection. In hosts with functional immune systems, the growth of these organisms is repressed and few pathological symptoms are observed. On the other hand, PCP is a cause of morbidity in HIV-positive patients as well as hosts with other immune defects, or in patients undergoing therapy with immunosuppressive agents [1–3]. Despite a decreased incidence of PCP in developed countries (due to the introduction of Highly Active Anti-Retroviral Therapy), the infection still causes death in about 15% of HIV-infected patients [4–6].

The genus *Pneumocystis* is comprised of many species, including *P. carinii, P. jirovecii* [7]*, P. wakefieldiae, P. murina* [8]*,* and *P. oryctolagi* [9–11]. These different species are characterized by their ability to infect different hosts. For example, *P. jirovecii* resides in the human lung alveoli. Despite their differences in host preference, all *Pneumocystis* species are hypothesized to have a bi-phasic life cycle: a) an asexual phase of replication via the binary fission of the trophic forms; b) a sexual phase in which the conjugation of trophic forms results in formation of asci which contain 8 ascospores, that are released and either continue in the sexual phase or enter the asexual phase [9].

Unlike mammalian cells, *Pneumocystis* is unable to harvest folate from the environment and must synthesize it de novo [12]. To take advantage of this weakness, the primary therapy for PCP is TMP-SMX, which inhibits dihydropteroate synthase and dihydrofolate reductase, the integral enzymes involved in folate synthesis in host cells and fungi [13–15]. Despite high success rates in treating PCP, TMP-SMX therapy leads to significant side effects, including neutropenia and serious allergic skin reactions that can result in death. It’s estimated that between 25 and 50% of HIV-infected patients are unable to tolerate prolonged TMP-SMX treatment due to these harsh side effects and must seek other treatment options [16].

Currently, alternative medications include atovaquone, clindamycin-primaquine, echinocandins, and pentamidine isethionate. Atovaquone inhibits nucleic acid and adenosine triphosphate synthesis [17], thus disrupting DNA replication, energy production, and proliferation of the fungi. A combination of clindamycin and primaquine suppresses fungal protein synthesis and mitochondrial function [18] i. The echinocandin family (i.e. anidulafungin, caspofungin, and micafungin) are β-1,3-D-glucan (BG) synthase inhibitors. Since BG is an essential component of the cellular wall that surrounds the asci of *Pneumocystis*, these drugs selectively target fungi in this phase [19–21]. The targets of the drug pentamidine isethionate remain unknown, although the drug has been shown to be effective [22]. When compared to TMP-SMX, these alternative therapies suffer from high rates of relapse and recurrence [23, 24]. Development of new drugs to treat PCP promises to deliver effective treatment with reduced side effects.

In comparison to other pathogens, the study of *Pneumocystis* is particularly challenged by the fact that these fungi cannot be reliably cultured in vitro for any significant length of time, nor continuously passaged to identify whether drugs are pneumocysticidal or pneumocystistatic. Due to this limitation*,* preclinical drug efficacy studies are carried out in animal models of *Pneumocystis* infection, typically in mice or rats [25]. Such reliance on animal studies significantly increases both the time and costs associated with the development of treatments to combat PCP. To alleviate this, it will be beneficial to integrate currently available knowledge on the treatment of PCP and our current knowledge of the *Pneumocystis* lifecycle into a QSP model to facilitate the drug development process. By combining traditional PK and PD analysis with systems biology modeling, QSP can summarize available information into a convenient framework, which can then be used to rigorously test different hypotheses, and scan through treatment regimens in an efficient and cost-effective manner [26, 27]. QSP modeling has been useful in the treatment of infectious diseases, such as Tuberculosis, where it has been used for dose optimization of anti-Tuberculosis drugs [28–30]. In addition, QSP models have shown great promise as powerful quantitative tools to study the dosing regimens for novel compounds [31].

A QSP model for the treatment of *Pneumocystis* is not yet available, and the scarcity of data from human patients makes the development of a human model difficult. With available data in mice, we constructed and validated a QSP model of PCP. This model includes both a PK module and a PD module. The PK module describes the distribution and decay of an applied drug, with different drugs characterized by their respective rate constants. This module was parametrized using independent construction and validation datasets. Following validation, the model was then used to predict the temporal PK profiles of standard dosing regimens in mice.

The PD module specifies the proliferation, transformation, and death of *Pneumocystis in* infected mice*.* The PK module and PD modules were then integrated into a population of QSP models. The parameters of this integrated model were estimated using a population of models that recapture the steady state distributions of the trophic forms and asci following drug treatment. The temporal dynamics generated by these QSP models were further validated with the observed dynamics of *Pneumocystis* following these same drug treatments.

After constructing independent PK and PD modules with data from various literature sources, the independent modules were then integrated to form a QSP model which was further validated using novel data of the temporal dynamics of *Pneumocystis* infection. As result, the QSP models developed in this work promise to serve as a solid first step towards understanding the temporal dynamics of *Pneumocystis* infection and facilitating the design of novel therapies. In the future, this model can potentially be improved and projected to a human version.

## Methods

**Fig. 1:**
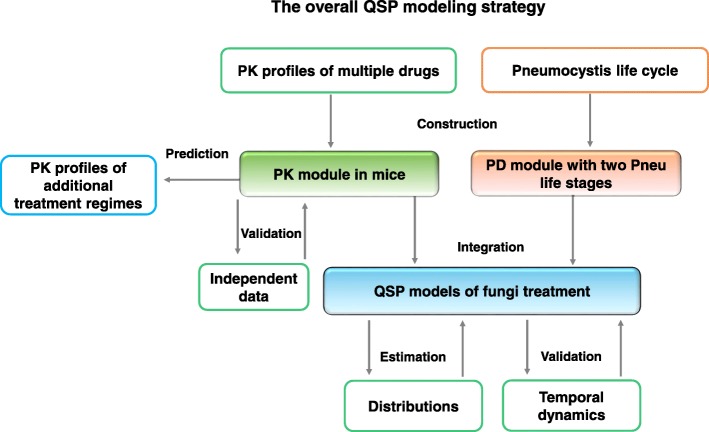
The overall QSP modeling strategy. The constructed QSP model includes both a PK module and a PD module. The PK module describes the distribution and decay of different drugs. The PD module specifies the proliferation, transformation, and death of the trophic forms and asci of *Pneumocystis* fungi. After construction of the PK module, this module was validated with independent data that were not used for its construction. For the PD module, all available data were used for its construction. The integrated QSP model, which includes both the PK module and the PD module, was constructed with the distribution of asci and trophic forms following treatment and then validated with their temporal dynamics

Our overall modeling strategy is illustrated in Fig. 1. After a PK module and a PD module were constructed, they were integrated into a comprehensive QSP model.

### Construction of the PK module in mice

**Fig. 2:**
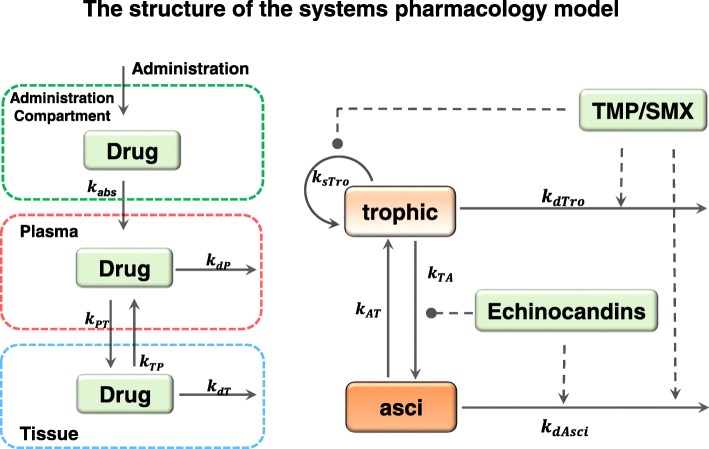
The structure of the QSP model. **Left panel:** A three-compartment PK module was used to describe the reported pharmacokinetic data. The first compartment was the AC, the second compartment was plasma, and the third was ‘peripheral tissue’. Drug decay was assumed to occur in plasma and ‘peripheral tissue’ compartments. The rates of drug distribution and decay were described by the corresponding parameters. **Right panel:** The dynamics of *Pneumocystis* were described by a two-stage model which involves both trophic forms and asci. The temporal changes of trophic forms and asci were also controlled by the indicated parameters. The drug effects were indicated by arrows (promoting) and lines with solid circle heads (inhibiting)

A three-compartment PK module was used to describe drug dynamics (Fig. 2). Drugs can be administrated either through intravenous (*i.v.*) injection, intraperitoneal (*i.p.*) injection or oral (*p.o.*) administration. In order to mimic *i.v.* injection, we elevated the initial level of the drug in the plasma compartment. To model *i.p*. or *p.o* treatments, drug was added to the administration compartment (AC) (Fig. 2). The level of the drug first increases in the AC, then diffuses into the plasma compartment. In this way, we were able to constrain the PK module with data from sources that administrated drugs via multiple methods.

Overall the module is comprised of the AC, a plasma compartment and a “*peripheral tissue*” compartment (combining all organs, muscles and fat *etc*).

Drug decay is assumed to occur in both the plasma and “peripheral tissue” compartments. The parameters that govern drug distribution and decay are labeled near the corresponding reaction arrows (Fig. 2).

The PK module was constructed with previously reported pharmacokinetic profiles (references elaborated in Table 2). Using figures from these works that plot drug concentration against time, digital values were extracted with the publicly available software labnotes (http://mpf.biol.vt.edu/lab_website/Labnotes.php).Three ordinary differential equations (ODEs) with identical structures (detailed below) were used to describe all drugs, while the rate constants differ between individual drugs. (Table 1).

**Table 1:** The equations and parameters of the PK module

Absorptive Compartment				
$$ \frac{{\boldsymbol{dDrug}}_{\boldsymbol{AC}}}{\boldsymbol{dt}}=-\boldsymbol{RAP}\ast {\boldsymbol{K}}_{\boldsymbol{abs}}\ast {\boldsymbol{Drug}}_{\boldsymbol{AC}} $$				
Plasma Compartment				
$$ \frac{{\boldsymbol{dDrug}}_{\boldsymbol{P}}}{\boldsymbol{dt}}={\boldsymbol{K}}_{\boldsymbol{abs}}\ast {\boldsymbol{Drug}}_{\boldsymbol{AC}}-\left({\boldsymbol{K}}_{\boldsymbol{P}\boldsymbol{T}}+{\boldsymbol{K}}_{\boldsymbol{dP}}\right)\ast {\boldsymbol{Drug}}_{\boldsymbol{P}}+{\boldsymbol{K}}_{\boldsymbol{T}\boldsymbol{P}}\ast {\boldsymbol{Drug}}_{\boldsymbol{T}} $$				
Peripheral Tissue Compartment				
$$ \frac{{\boldsymbol{d}\boldsymbol{Drug}}_{\boldsymbol{T}}}{\boldsymbol{d}\boldsymbol{t}}=\boldsymbol{RTP}\ast \left(-{\boldsymbol{K}}_{\boldsymbol{T}\boldsymbol{P}}\ast {\boldsymbol{Drug}}_{\boldsymbol{T}}+{\boldsymbol{K}}_{\boldsymbol{P}\boldsymbol{T}}\ast {\boldsymbol{Drug}}_{\boldsymbol{P}}\right)-{\boldsymbol{K}}_{\boldsymbol{d}}\ast {\boldsymbol{Drug}}_{\boldsymbol{T}} $$				
PK parameters for each drug				
	**anidulafungin**	**caspofungin**	**micafungin**	**TMP/ SMX**
***K***_***dP***_ (hr ^− 1^)	0.035	0.18	0.06	0.2
***K***_***PT***_ (hr ^−1^)	1.5	5	0.6	0.17
***K***_***TP***_ (hr ^−1^)	5	2	1.8	5
***RTP*** *(dimensionless)*	0.2	1	0.4	0.01
***K***_***dT***_ (hr ^−1^)	0.035	0.18	0.06	0.2
***RAP*** *(dimensionless)*	3	0.1	1	3
***K***_***abs***_ (hr ^−1^)	5	5	0.75	5

### The drug concentration in the administration compartment (AC) is modeled as:

$$ \frac{\boldsymbol{dDrugAC}}{\boldsymbol{dt}}=-\boldsymbol{RAP}\ast {\boldsymbol{K}}_{\boldsymbol{abs}}\ast {\boldsymbol{Drug}}_{\boldsymbol{AC}} $$

Where ***Drug***_***AC***_ represents the current level of Drug in the Absorptive compartment, ***K***_***abs***_ represents the absorption rate of the drug, and ***RAP*** is a non-dimensional scaling factor. Since the drug resides in the administration compartment for only a short time, its decay is not explicitly incorporated.

**The drug concentration in the plasma compartment is modeled as:**

$$ \frac{\boldsymbol{dDrugP}}{\boldsymbol{dt}}={\boldsymbol{K}}_{\boldsymbol{abs}}\ast {\boldsymbol{Drug}}_{\boldsymbol{AC}}-\left({\boldsymbol{K}}_{\boldsymbol{P}\boldsymbol{T}}+{\boldsymbol{K}}_{\boldsymbol{dP}}\right)\ast {\boldsymbol{Drug}}_{\boldsymbol{P}}+{\boldsymbol{K}}_{\boldsymbol{T}\boldsymbol{P}}\ast {\boldsymbol{Drug}}_{\boldsymbol{T}} $$

Where ***Drug***_***P***_ represents the current level of Drug in the Plasma compartment, ***K***_***abs***_ represents the absorption rate of the drug, ***K***_***PT***_the rate at which drug moves from the Plasma compartment to the Tissue compartment, ***K***_***d***_ is the degradation rate of the Drug and ***K***_***TP***_ is the rate at which the Drug moves from the tissue compartment to the plasma compartment.

**The drug concentration in the peripheral tissue compartment is modeled as:**

$$ \frac{\boldsymbol{d}\boldsymbol{DrugT}}{\boldsymbol{d}\boldsymbol{t}}=\boldsymbol{RTP}\ast \left(-{\boldsymbol{K}}_{\boldsymbol{T}\boldsymbol{P}}\ast {\boldsymbol{Drug}}_{\mathbf{T}}+{\boldsymbol{K}}_{\boldsymbol{P}\boldsymbol{T}}\ast {\boldsymbol{Drug}}_{\boldsymbol{P}}\right)-{\boldsymbol{K}}_{\boldsymbol{d}}\ast {\boldsymbol{Drug}}_{\boldsymbol{T}} $$

Where ***Drug***_***T***_ represents the current level of Drug in the tissue compartment and ***RTP*** represents a non-dimensional scaling factor. The values of the dimensionless factors **RAP** and **RTP** are estimated from the observed data for each drug.

For the PK modules, all parameters sets and initial conditions were derived manually using a trial and error method to find a plausible parameter set that visually recaptures the experimentally observed data. The initial conditions for each drug were estimated from the literature data when available. For example, the initial level of Anidulafungin was estimated to be 90 μg/ml for the i.p. dosage of 10 mg/kg (34). When such data was not available, higher or lower initial levels were assumed for higher or lower dosage of applied drug. The sum of squared error (SSE) for each parameter set were calculated and summarized (Table 2).

**Table 2:** The experimental data for the construction and validation of PK modules

Drug	anidulafungin	caspofungin	micafungin	TMP/ SMX
Construction data	Gumbo et al.*.*. [35]	Andes et al. [40]	Andes et al. [40]	Misiek et al. [41]
Validation data	Andes et al [42]	Andes et al. [40]; Hajdu et al. [43]	Andes et al. [40];	Misiek et al. [41]
Goodness of fit (SSE)	459.26	2602.56	202.14	131.15

### Construction of the PD module in mice

The life cycle of *Pneumocystis*, including its proliferation, life cycle stage transformation, and death, was simplified into a two-stage model which included both trophic forms and asci (Fig. 2). The simplified model was described using a pair of ODEs and 5 control parameters.

Trophic forms of the organism were model by the following ODE:

$$ \frac{\boldsymbol{dTro}}{\boldsymbol{dt}}={\boldsymbol{K}}_{\boldsymbol{sTro}}\ast \boldsymbol{Tro}-{\boldsymbol{K}}_{\boldsymbol{dTro}}\ast \boldsymbol{Tro}\ast \boldsymbol{Tro}-{\boldsymbol{K}}_{\boldsymbol{TA}}\ast \boldsymbol{Tro}+{\boldsymbol{K}}_{\boldsymbol{AT}}\ast \boldsymbol{Asci} $$

Where *Tro* represents the current value of the Trophic form of *Pneumocystis*, *K*_*sTro*_ is the proliferation rate of ***Tro*,** ***K***_***dTro***_ is the death rate of ***Tro***, ***K***_***TA***_ is the rate at which trophic forms are converted to asci, and ***K***_***AT***_ represents the rate at which asci are converted to trophic forms.

Asci were described by the following ODE:

$$ \frac{\boldsymbol{dAsci}}{\boldsymbol{dt}}={\boldsymbol{K}}_{\boldsymbol{TA}}\ast \boldsymbol{Tro}-{\boldsymbol{K}}_{\boldsymbol{AT}}\ast \boldsymbol{Asci}-{\boldsymbol{K}}_{\boldsymbol{dAsci}}\ast \boldsymbol{Asci} $$

Where ***Asci*** represents the current value of the asci of the fungi, and ***K***_***dAsci***_ represents its death rate.

Our model describes the transformation between trophic forms and asci following a similar multistate model of tuberculosis [32]. Following logistic growth models, the decay of the trophic form is a second order reaction since the trophic forms actively proliferate and compete for space and nutrients. On the contrary, the asci do not actively proliferate but rather result from the transformation of trophic forms. Hence, the decay of the asci is set to be a first order reaction.

The basal values of these control parameters were estimated on the basis of relevant experimental data (Table 4).

**Fig. 3:**
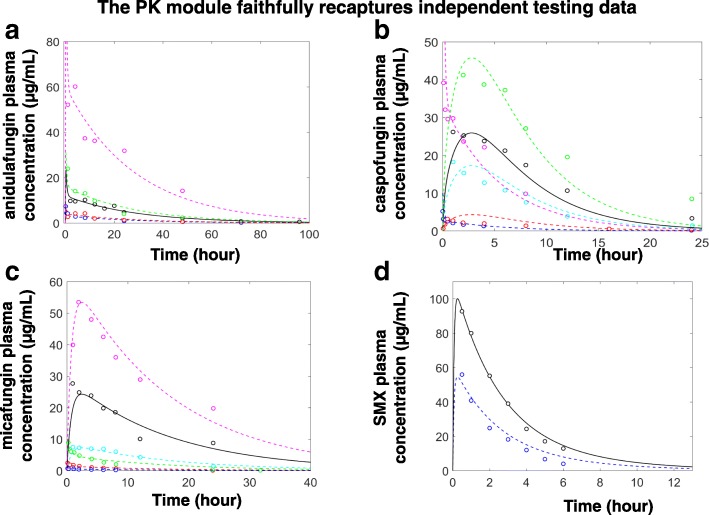
The PD modules were consistent with experimental data from diverse sources. **a**. Temporal simulations for the dynamic changes of trophic form (black curves) and asci (red curves) starting from an initial state with a high level of trophic forms and a low level of asci. **b**. Temporal simulations (black curves) of the normalized total number of *Pneumocystis* were compared to the normalized nuclei count from *Pneumocystis* infected mice (red dots, error bars represent SEM, *n* = 2 or 3 for each time point). **c** and **d**. Histograms showing the distributions of the numbers of the trophic form and asci simulated by the PD module

**Fig. 4:**
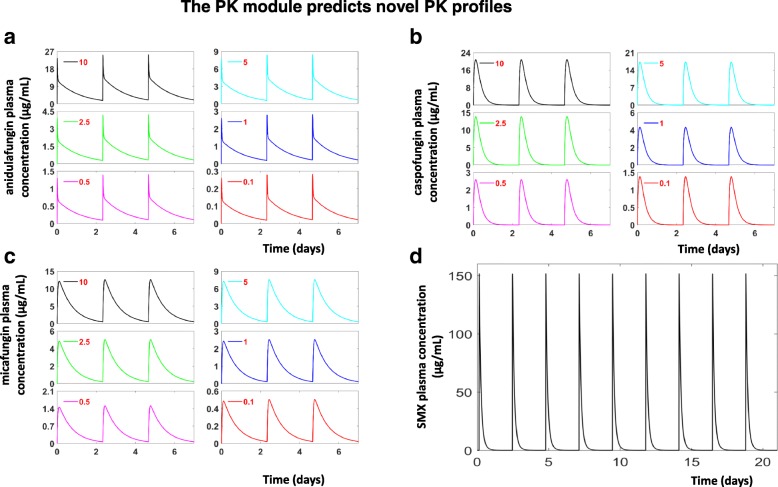
The simulations of the QSP models were consistent to relevant data. **a** and **b**. Bar plots of average simulated log_10_ levels: of asci (**a**) and trophic forms (**b**) at day 56 post-treatment of *Pneumocystis* from: untreated mice (Control), mice treated with varying doses of anidulafungin, caspofungin and micafungin; as well as mice treated with TMP-SMX. Corresponding experimental data are represented as dot plots with standard error. **c**. The simulated dynamic changes of the trophic forms (black curves) and asci (red curves), on a log_10_ scale were consistent to the corresponding experimental data (black and red dots) following anidulafungin treatment. **d**. The simulated dynamic changes of trophic forms (black curves) and asci (red curves) were consistent to the corresponding data (black dots and red dots) following TMP-SMX treatment

The experimentally observed levels of *Pneumocystis* (Figs. 3 & 4) are distributed over a broad range. To recapture these experimentally observed distributions, we constructed a population of PD models with parameter values selected from a uniform distribution that covers 70–130% of the basal values (Table 4). For Fig. 3b, the experimental results are reported as a total nuclei count of both trophic forms and asci that is on a different scale than the PD model. To recapture this dataset, the model results were converted into a nuclei count and rescaled to the maximum.

### Integration of the PK module and the PD modules into QSP models

Various drugs target *Pneumocystis* via diverse mechanisms, which were incorporated into the QSP models. TMP-SMX represses folate synthesis which is essential for genome replication in the organism [13]. Therefore, in our simplified model, TMP-SMX was assumed to inhibit the proliferation rate of the trophic forms and increase the death rates of both the trophic forms and asci. Echinocandins on the other hand, block the construction of the cellular wall of the asci. Therefore, this family of drugs were assumed to reduce the level of asci by promoting their death as well as inhibiting their formation (Fig. 2 and Table 5).

The EC50 and maximal effect of each drug were estimated from the levels of asci and trophic forms following treatment with different drugs. Since the ratio of TMP-SMX was fixed to be 1:5 in the data constraining our QSP model, we simplified the model by using the level of SMX as a reasonable proxy for this drug combination.

To account for the drug effects on pneumocystis in the QSP models, we replaced the constant parameters of the PD modules (**k**_**s**_, **k**_**dTro**_, **k**_**dAsci**_ and **k**_**TA**_) with corresponding functions of the levels of drugs (**v**_**sTro**_, **v**_**dTro**_, **v**_**dAsci**_, and **v**_**TA**_,Table 5) .

In the presence of SMX, the death rates of both the trophic forms and asci are enhanced and descried with the following equation:

$$ {\mathbf{v}}_{\mathbf{dTro}}={\mathbf{k}}_{\mathbf{dTro}}\ast \left(\mathbf{1}+{\mathbf{ME}}_{\mathbf{Tro}}\ast \frac{{\mathbf{SMX}}_{\mathbf{eff}}^{\mathbf{n}}}{{\mathbf{SMX}}_{\mathbf{eff}}^{\mathbf{n}}+\mathbf{Ec}{\mathbf{50}}_{\mathbf{SMX}}^{\mathbf{n}}}\right) $$

where **k**_**dTro**_, represents the basal death rate of the trophic form, **ME**_**Tro**_ is the maximal effect by SMX, **SMX**_**eff**_ is the effective level of SMX, **EC50**_**SMX**_ is the half maximal effective concentration of SMX, and n is the hill coefficient . A similar equation is used to calculate, **v**_**dAsci**_, the death rate of the asci when exposed to SMX.

The presence of SMX also leads to repression of tro proliferation, which is described with the following equation:

$$ {\mathbf{v}}_{\mathbf{s}\mathbf{Tro}}={\mathbf{k}}_{\mathbf{s}}\ast \left(\mathbf{1}-\frac{{\mathbf{SMX}}_{\mathbf{eff}}^{\mathbf{n}}}{{\mathbf{SMX}}_{\mathbf{eff}}^{\mathbf{n}}+\mathbf{Ec}{\mathbf{50}}_{\mathbf{SMX}}^{\mathbf{n}}}\right) $$

Where **k**_**s**_ represents the basal proliferation rate of the trophic form.

The presence of echinocandins inhibits the asci specifically. To incorporate its effect, we assume that the transformation of trophic forms to asci is inhibited and that the death rate of the asci is enhanced. The enhanced death of asci is modeled with a similar equation as replaced above. The inhibition of asci formation is described with the following equation:

$$ {\mathbf{v}}_{\mathbf{TA}}={\mathbf{k}}_{\mathbf{TA}}\ast \left(\mathbf{1}-\frac{{\mathbf{Echi}}_{\mathbf{pla}}^{\mathbf{n}}}{{\mathbf{Echi}}_{\mathbf{pla}}^{\mathbf{n}}+\mathbf{Ec}{\mathbf{50}}_{\mathbf{Echi}}^{\mathbf{n}}}\right) $$

Where **k**_TA_ represents the normal rate of asci formation, while **Echi**_**pla**_ is the current plasma level of echinocandin*.*

#### Software

The ordinary differential equations were simulated with the mathematical software XPPAUT, which is freely available at http://www.math.pitt.edu/~bard/xpp/xpp.html. The experimental and simulated data were then visualized using MATLAB from Mathworks (https://www.mathworks.com).

### Experimental methods

#### Measuring pneumocystis numbers in mice

The *Pneumocystis* number is commonly estimated in two ways: reverse Transcriptase quantitative PCR (RT-qPCR) or microscopic quantification.

For all mouse studies, 6 week old male, C3H/HeN mice were used. These came from the animal supply company Charles River (*https://www.criver.com*).

For RT-qPCR, *Pneumocystis*-infected mice were euthanized by CO2 exposure until cessation of breathing at regular intervals and the lungs flash frozen, followed by RNA extraction and cDNA synthesis. *Pneumocystis* mitochondrial large subunit ribosomal RNA, was then quantified by TaqMan assay. The threshold cycle for each sample was identified as the point at which the fluorescence generated by degradation of the TaqMan probe increased significantly above the baseline. To convert the threshold cycle to *Pneumocystis* nuclei number, a standard curve was generated using cDNA made from RNA isolated from 10^7^ *Pneumocystis* nuclei. The level of infection for each sample was estimated using the standard curve [33].

Although accurate, this technique cannot distinguish between the trophic forms and asci of *Pneumocystis*.

For microscopic quantification with a use a Nikon Eclipse E600, lungs from *Pneumocystis*-infected mice were isolated and stained with a dye that selectively binds to the asci of the fungi, cresyl echt violet. A rapid version of the Wright-Giemsa stain was used to enumerate the nuclei of all life cycle stages [34]. In contrast to RT-qPCR, microscopic quantification allows for the distinction between the trophic forms and asci.

Though these methods rely on different techniques, the time scale characterizing *Pneumocystis* is independent of the method used. This common time scale facilitated the construction of the current PD modules with both literature reported numbers of trophic forms and asci and novel experimental results using the RT-qPCR method.

## Results

### The constructed PK module was validated against independent data

**Fig. 5:**
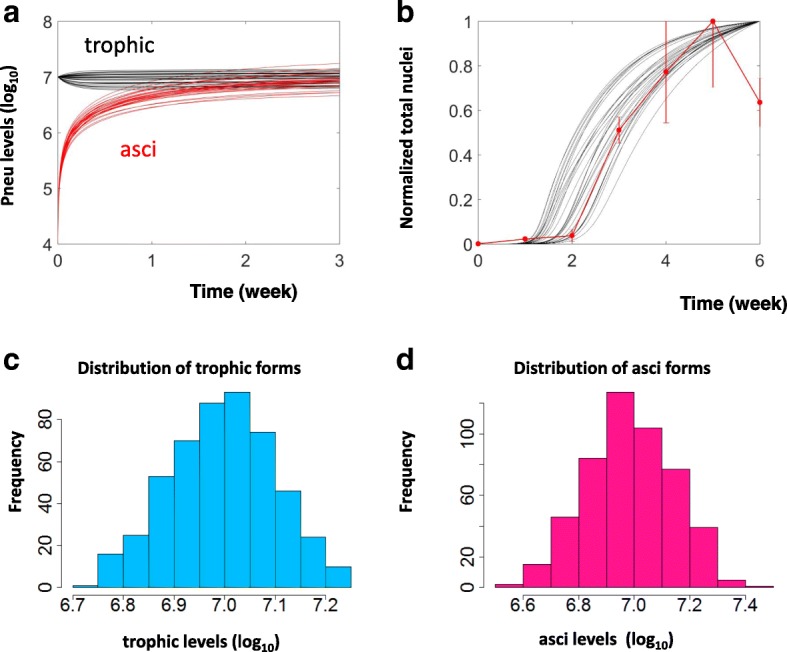
The temporal simulations of the PK modules were consistent with diverse experimental data. The temporal simulations of the plasma concentrations of anidulafungin (**a**), caspofungin (**b**), micafungin (**c**) and smx (**d**) were compared to relevant experimental data. The black dots and black solid curves represent the construction data and corresponding model simulations; the colored dots and colored dashed curves represent the validation data and corresponding simulations. The data sources were elaborated in Table 2. The colors in each panel were used to indicate different administration methods and dosages. In **a**, blue, *i.v*. of 1 mg/kg; magenta, green and red, *i.p.* of 80 mg/kg, 20 mg/kg and 5 mg/kg respectively. In **b**, blue and magenta, *i.v*. of 0.5 mg/kg and 5 mg; red, cyan and green, *i.p.* of 1 mg/kg, 5 mg/kg and 80 mg/kg; In **c**, blue, red and green, *i.v*. of 0.32 mg/kg, 1 mg/kg and 3.2 mg/kg; cyan and magenta, *i.p.* of 5 mg/kg and 80 mg/kg; In **d**, blue, oral of 50 mg/kg

The equations and parameter values of the constructed PK modules were reported in Table 1, with each drug characterized by a different set of parameter values. These parameter values were estimated using data reported in the literature (Table 2). For example, Gumbo et al. measured the plasma concentration of anidulafungin following a single 10 mg/kg i.p. injection [35], which we used to estimate the PK parameters for a three compartment pharmacokinetic model of anidulafungin. (Fig. 5a). After estimating the PK parameters, they were used to simulate further experimental scenarios with either *i.p.* or *i.v.* administration of anidulafungin and compared to their respective data sets (Fig. 5a**)**. Because these additional data sources were not used for the initial parameter estimation, the consistency between the model simulation and these additional data sources served as a validation of the estimated parameters for the anidulafungin PK model.

In a similar fashion, the parameters for PK models of caspofungin, micafungin, and SMX were estimated and validated with different literature sources (Fig. 5b-d**)**.

### The PK modules predict novel PK profiles

Once constructed and validated, our PK modules may serve as convenient tools to predict the plasma level of each drug following more than a single dose. To illustrate this potential, we used the PK modules to predict the plasma levels of four different drugs following the reported treatment regimens [33].

**Fig. 6:**
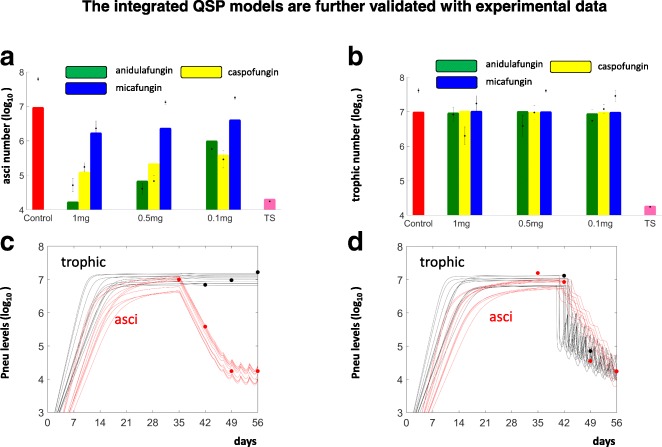
The temporal drug profiles predicted by the PK modules. **a**, **b**, **c** and **d** show the predicted plasma levels of anidulafungin, caspofungin, micafungin and SMX when administrated 3 times/week. The different dosages of anidulafungin, caspofungin, micafungin (in mg/kg) are labelled in each panel, the SMX dosage is 200 mg/kg

Here, three drugs from the echinocandin family (anidulafungin, caspofungin and micafungin) were administrated through *i.p.* injection and a fourth drug, TMP-SMX, was administered orally [33]. Given the reported dosage of each treatment, we estimated the expected increases of each drug in the administration compartment, which served as the in silico drug dosage (Table 3). Following the experimental dosing regimen reported by Cushion et al., each drug was elevated three times a week for 3 weeks [33]. The simulated plasma levels of echinocandins within one week and SMX within three weeks were shown in Fig. 6.

**Table 3:** Estimated Initial AC concentrations of echinocandins and SMX for model prediction

Applied dosage (mg/kg)	Initial Anidulafungin concentration (μg**/**ml)	Initial Caspofungin concentration (μg**/**ml)	Initial Micafungin concentration (μg/ml)
**10**	90 based on Data in [35]	12	25
**5**	30 based on Data in [42]	10 based on Data in [40]	15 based on the Data in [40]
**2.5**	15	8	10
**1**	10	2.5	5
**0.5**	5	1.5	3
**0.1**	1	0.8	1

* For SMX, 500 and 550 (μg/ml) were used for applied dosages of 200 and 250 (mg/kg) respectively

Compared with traditional pharmacokinetic indexes such as area under the curve, the temporal predictions from the PK modules elaborated temporal dynamics of applied drugs that might play a significant role in determining drug effectiveness [36]. When additional PK data are available, these data can be used to further refine the PK modules and reduce the need of repeating PK measurements.

### The constructed PD modules were consistent with multiple experimental observations

After the PD wiring diagram (Fig. 2) was converted into ODEs (details in methods), the parameters of the module were estimated with currently available data (Table 4). In order to check whether the estimated parameters are reasonable, the temporal simulations of the PD module were compared to these experimental observations.

**Table 4:** The equations and parameters of the PD modules

trophic Form		
$$ \frac{\boldsymbol{dTro}}{\boldsymbol{dt}}={\boldsymbol{K}}_{\boldsymbol{sTro}}\ast \boldsymbol{Tro}-{\boldsymbol{K}}_{\boldsymbol{dTro}}\ast \boldsymbol{Tro}\ast \boldsymbol{Tro}-{\boldsymbol{K}}_{\boldsymbol{TA}}\ast \boldsymbol{Tro}+{\boldsymbol{K}}_{\boldsymbol{AT}}\ast \boldsymbol{Asci} $$		
asci		
$$ \frac{\boldsymbol{dAsci}}{\boldsymbol{dt}}={\boldsymbol{K}}_{\boldsymbol{TA}}\ast \boldsymbol{Tro}-{\boldsymbol{K}}_{\boldsymbol{AT}}\ast \boldsymbol{Asci}-{\boldsymbol{K}}_{\boldsymbol{dAsci}}\ast \boldsymbol{Asci} $$		
Basal Parameter Values		
Parameter	Value (unit)	Constraining data
***K***_***sTro***_	1 day ^− 1^	The observed accumulation of total *Pneumocystis* constrained the time scale, The steady state values of trophic form constrained the *K*_*sTro*_ : *K*_*dTro*_ ratio.
***K***_***dTro***_	1 × 10^−7^ day ^− 1^	
***K***_***AT***_	0.1 day ^− 1^	The *K*_*TA*_ : *K*_*AT*_ ratio was constrained with experimental observations [33]
***K***_***TA***_	0.1 day ^− 1^	
***K***_***dAsci***_	2 × 10^− 12^ day ^− 1^	Degradation rate of the asci is assumed to be small [44].

By specifically targeting the asci of the fungi, administration of the anti-fungal drug anidulafungin can result in a state with a low level of asci and a high level of trophic forms. Starting from this initial state, and in the absence of any drug treatment, it takes several weeks for asci to repopulate [33]. The time range of this recovery was consistent to a number of temporal simulations of the PD module (Fig. 3a) with initial conditions that mimicked this experimental scenario. The consistency between time frames suggests that the estimated rates characterizing the transformation from the trophic form to asci (***K***_***TA***_) fall within a biologically reasonable scale.

In addition to literature reported data, we have also experimentally determined the total number of *P. murina* nuclei within infected and immunosuppressed mice (red dots, Fig. 3b). The initial growth of the organism was very slow within the first two weeks, however, starting from the third week, an exponential growth of *Pneumocystis* was observed which peaked at the end of the fifth week. The experimentally determined nuclei count was then compared with a population of simulated temporal curves of *Pneumocystis* accumulation (Fig. 3b). The model simulations recaptured the slow initial accumulation of the *Pneumocystis*, the rapid, exponential growth of the organism, and the steady state level following the exponential peak. The consistency between this experimental data and the temporal simulations suggests that the model assumption of rapid *Pneumocystis* growth is indeed reasonable.

The level of *Pneumocystis* begins to decrease near the end of the experiment (red outlier, Fig. 3b). This is likely due to depletion of nutrients or overcrowding. Since these mechanisms have not been incorporated into the current model, it is not surprising that the model simulations fail to recapture the observed decrease.

Furthermore, the simulated distributions of the trophic forms (Fig. 3c) and asci (Fig. 3d) are consistent to the observed levels of the fungi (7.62 ± 0.17 for trophic forms and 7.79 ± 0.13 for asci) [33]. The agreement between the experimentally determined pneumocystis level and those simulated with the model, suggests that the assumed ratios between proliferation and decay (ratio between ***K***_***sTro***_ and***K***_***dTro***_) and transformation (ratio between ***K***_***TA***_and ***K***_***AT***_) are reasonable. In order to incorporate variability, all model parameters are changed independently.

### Quantitative systems pharmacology model construction and validation

By integrating the PK modules and the PD module, the QSP model can describe the changes of asci and trophic forms following treatment for a population of models. Modules were integrated by adjusting the parameters that control: the growth and death of the cyst form (for the echinocandin family of drugs, Fig. 2), or the death rates of the trophic and cyst forms along with the growth of the trophic form (for TMP/SMX treatment, Fig. 2). Details of the integration procedure can be found in the **Methods**. Following the experimental setting as reported by Cushion et al., each drug in the model was administrated 3 times per week for 3 weeks [33]. The simulated levels of asci at day 56 were then compared to the experimental observations from Cushion et al. (Fig. 4a). At a dose of 1 mg/kg, treatment with all three echinocandins (anidulafungin, caspofungin and micafungin) considerably reduced asci burdens. At lower doses (0.5 and 0.1 mg/kg), anidulafungin and caspofungin still decreased the number of asci, while micafungin caused no notable decrease in the levels of asci (Fig. 4a). In contrast to the dramatic reductions in asci, the simulated trophic forms were not meaningfully altered following treatment with any of the echinocandins (Fig. 4b). The model showed a marked decrease in both asci and trophic forms in response to TMP/SMX treatment (Fig. 4a & b). These simulated results were consistent with the experimental observations [33], indicating that our integrated QSP models are reasonable in describing the therapeutic effects of the echinocandin family of drugs and those of TMP/SMX.

With the constructed QSP models, we then simulated the temporal changes of asci and trophic forms prior to and after anidulafungin treatment (Fig. 4c). Prior to drug administration, the simulated accumulation of both trophic forms and asci are consistent to experimental data collected in the absence of drugs, as elaborated in the description of the PD modules above. At about 35 days, the levels of both trophic forms and asci reached a steady state of about 10^7^, in agreement to the experimental data (Fig. 4c). Following anidulafungin treatment (starting at day 35), the level of asci decreased dramatically while the level of trophic form remained constant. These simulated responses to anidulafungin were consistent with the corresponding experimental data from our lab (Fig. 4c).

When compared to anidulafungin, treatment with TMP-SMX decreased the levels of both asci and trophic forms. However, in comparison with the rapid antifungal effect of anidulafungin, the experimental evidence suggests that the effect of TMP-SMX was delayed. This time delay was incorporated into our QSP model (Table 5), and the simulated responses of trophic and asci levels (Fig. 4d) were consistent to corresponding experimental data (Fig. 4d). In summary, the QSP models serve as a reasonable tool to describe the temporal dynamics of Pneumocystis upon treatment with either the echinocandin class of antifungals or TMP/SMX.

**Table 5:** Integrating the PK modules and PD modules into QSP models

Echinocandin effect on asci death				
$$ {\boldsymbol{v}}_{\boldsymbol{dAsci}}={\boldsymbol{k}}_{\boldsymbol{dAsci}}+{\boldsymbol{ME}}_{\boldsymbol{Echi}}\ast \frac{{\boldsymbol{Echi}}_{\boldsymbol{pla}}^{\boldsymbol{n}}}{{\boldsymbol{Echi}}_{\boldsymbol{pla}}^{\boldsymbol{n}}+\boldsymbol{Ec}{\mathbf{50}}_{\boldsymbol{Echi}}^{\boldsymbol{n}}} $$				
Echinocandin effect on asci Formation				
$$ {\boldsymbol{v}}_{\boldsymbol{TA}}={\boldsymbol{k}}_{\boldsymbol{TA}}\ast \left(\mathbf{1}-\frac{{\boldsymbol{Echi}}_{\boldsymbol{pla}}^{\boldsymbol{n}}}{{\boldsymbol{Echi}}_{\boldsymbol{pla}}^{\boldsymbol{n}}+\boldsymbol{Ec}{\mathbf{50}}_{\boldsymbol{Echi}}^{\boldsymbol{n}}}\right) $$				
Parameters				
**Echinocandin family member**	***Ec*** **50**	***ME***	***n***	
Anidulafungin	0.039 μg ∗ ml^−1^	0.42	1	
Caspofungin	0.0007 μg ∗ ml^−1^	0.45	1	
Micafungin	0.04 μg ∗ ml^−1^	0.1	1	
TMP/SMX effect on asci death				
$$ {\boldsymbol{v}}_{\boldsymbol{dAsci}}={\boldsymbol{k}}_{\boldsymbol{dAsci}}+{\boldsymbol{ME}}_{\boldsymbol{Asci}}\ast \frac{{\boldsymbol{SMX}}_{\boldsymbol{eff}}^{\boldsymbol{n}}}{{\boldsymbol{SMX}}_{\boldsymbol{eff}}^{\boldsymbol{n}}+\boldsymbol{Ec}{\mathbf{50}}_{\boldsymbol{SMX}}^{\boldsymbol{n}}} $$				
TMP/SMX effect on trophic proliferation				
$$ {\boldsymbol{v}}_{\boldsymbol{s}\boldsymbol{Tro}}={\boldsymbol{k}}_{\boldsymbol{s}}\ast \left(\mathbf{1}-\frac{{\boldsymbol{SMX}}_{\boldsymbol{eff}}^{\boldsymbol{n}}}{{\boldsymbol{SMX}}_{\boldsymbol{eff}}^{\boldsymbol{n}}+\boldsymbol{Ec}{\mathbf{50}}_{\boldsymbol{SMX}}^{\boldsymbol{n}}}\right) $$				
TMP/SMX effect on trophic death				
$$ {\boldsymbol{v}}_{\boldsymbol{dTro}}={\boldsymbol{k}}_{\boldsymbol{dTro}}\ast \left(\mathbf{1}+{\boldsymbol{ME}}_{\boldsymbol{Tro}}\ast \frac{{\boldsymbol{SMX}}_{\boldsymbol{eff}}^{\boldsymbol{n}}}{{\boldsymbol{SMX}}_{\boldsymbol{eff}}^{\boldsymbol{n}}+\boldsymbol{Ec}{\mathbf{50}}_{\boldsymbol{SMX}}^{\boldsymbol{n}}}\right) $$				
Delay in SMX effect				
${\boldsymbol{SMX}}_{\boldsymbol{eff}}^{\boldsymbol{n}}$= ${\boldsymbol{SMX}}_{\boldsymbol{pla}}^{\boldsymbol{n}}\left(\boldsymbol{t}-\boldsymbol{\tau} \right)$				
Parameters				
$\boldsymbol{Ec}{\mathbf{50}}_{\boldsymbol{SMX}}^{\boldsymbol{n}}$	***MEAsci***	***METro***	***n***	***τ***
*0.2 μ****g*** ∗ ***ml***^−**1**^	0.75	650	2	7 days

## Discussion

In this work, we developed a QSP model to simulate how the numbers of *Pneumocystis* are altered by commercially available echinocandins and TMP-SMX. In addition to describing the temporal dynamics of these drugs, this novel QSP model also incorporated two different life cycle stages of the infecting fungi. Since the different life stages are presumably conserved in a broad range of hosts, the QSP model would be useful for studying *Pneumocystis* infections in a number of hosts including humans.

QSP modeling, which integrates knowledge from pharmacology and systems biology, is emerging as a powerful approach in pharmaceutical development [37, 38]. To the encouragement of the QSP community, QSP modeling aided in studying the dosing regimens of a new biologic, NATPARA, in the regulatory domain [31]. Particularly, QSP modeling has been useful in aiding the treatment of infectious diseases, such as tuberculosis, where it has been used for dose optimization of anti-Tuberculosis drugs [28–30]. Moreover, QSP models have shown great promise as powerful quantitative tools to study the dosing regimen for novel pharmaceutical compounds [31]. Thus, it is worthwhile to carefully evaluate the power as well as limitations of QSP modeling.

The benefits of QSP modelling originate from its ability to integrate all available knowledge and data to predict the effect of novel treatment regimens. In this way, the modeling provides some guidance for choosing effective strategies and avoiding plans that might have little chance for success. In this way, QSP combines traditional PK/PD modeling with systems biological modeling and provides a more comprehensive picture than single indices such as steady state AUC [39]. In order to generate faithful predictions, both the PK and PD portions of the QSP models must be carefully constructed and independently validated. For the current QSP model, the PK module has been well constrained with the abundant data available in the literature, however the PD module needs to be further validated with additional dynamic data of the asci and trophic forms following treatment with different drugs as well as dynamic data of the growth of the organism prior to treatment. These additional data sources will either validate the model’s current parameter settings or allow for further refinement of the parameters.

The complexity and scope of the current model aim to achieve a balance between incorporation of mechanistic details and constraint by currently available data. When additional details become available, the current PD module can be expanded to include a more detailed description of the Pneumocystis life stages, while the PK module can be expanded to incorporate additional compartments, such as a lung compartment. Furthermore, the model can be tailored to investigate additional drugs such as atovaquone or clindamycin-primaquine.

The current model, constrained with data collected in mice, promises to serve as a useful framework to understand and predict the growth, death and drug response of Pneumocystis in human patients, assuming the conservation of Pneumocystis life stages between species. Such predictions of *Pneuomocystis* levels in human, being orthogonal to the observed symptoms, will provide valuable insight for the clinicians to understand the progression of the infection as well as its response to treatment.

## Conclusions

We developed and validated a QSP model that integrates available data and promises to facilitate the design of future therapies against PCP.

## Abbreviations

*AC*: Administration compartment
*BG*: β-1,3-D-glucan
*i.p.*: Intraperitoneal
*i.v.*: Intravenous
*ODEs*: Ordinary differential equations
*p.o.*: Oral administration
*PCP*: *Pneumocystis pneumonia*
*PD*: Pharmacodynamics
*PK*: Pharmacokinetics
*QSP*: Quantitative Systems Pharmacological
*RT-qPCR*: Reverse Transcriptase quantitative PCR
*TMP-SMX*: Trimethoprim-sulfamethoxazole
