# Correction to: A quantitative systems pharmacology (QSP) model for Pneumocystis treatment in mice

**DOI:** 10.1186/s12918-019-0708-9

**Published:** 2019-08-12

**Authors:** Guan-Sheng Liu, Richard Ballweg, Alan Ashbaugh, Yin Zhang, Joseph Facciolo, Melanie T. Cushion, Tongli Zhang

**Affiliations:** 1 Department of Pharmacology and Systems Physiology, College of Medicine, University of Cincinnati, 231 Albert Sabin Way, Cincinnati, OH 45267-0576 USA; 2 Department of Internal Medicine, College of Medicine, University of Cincinnati, Cincinnati, OH USA; 3 Division of Biostatistics and Epidemiology, Cincinnati Children’s Hospital Medical Center, Cincinnati, OH USA

**Correction to: BMC Syst Biol (2018) 12:77**

**https://doi.org/10.1186/s12918-018-0603-9**

It was highlighted that the original article [1] contained errors in the figures and their legends and by extension the in-text figure citations (Figs. 1, 2, 3, 4, 5 and 6). This Corrections article shows the correct figures and correct figure legends. This Correction article includes a Table showing the incorrect and correct figure citations (Table 1).

**Fig. 1:**
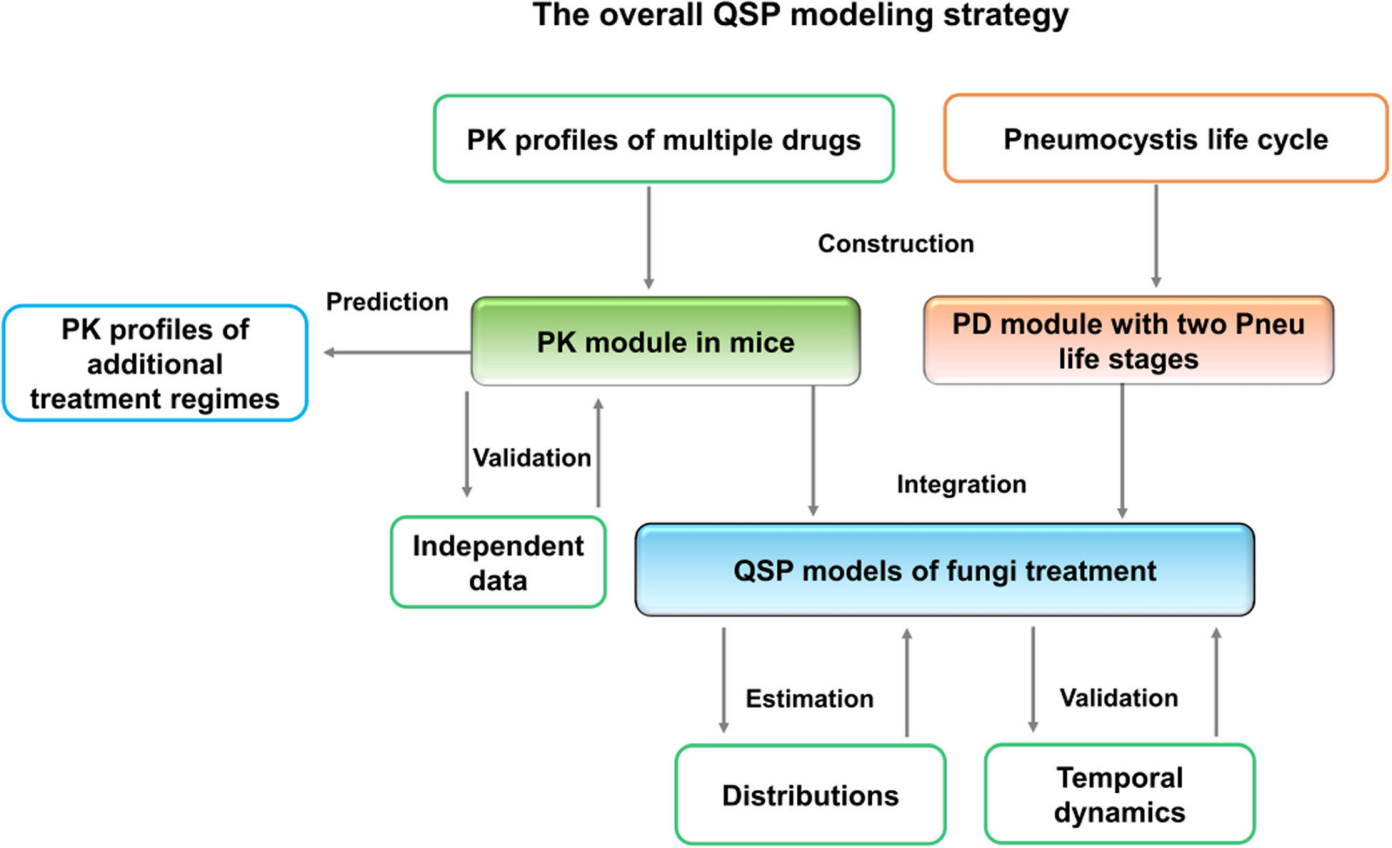
The overall QSP modeling strategy. The constructed QSP model includes both a PK module and a PD module. The PK module describes the distribution and decay of different drugs. The PD module specifies the proliferation, transformation, and death of the trophic forms and asci of *Pneumocystis* fungi. After construction of the PK module, this module was validated with independent data that were not used for its construction. For the PD module, all available data were used for its construction. The integrated QSP model, which includes both the PK module and the PD module, was constructed with the distribution of asci and trophic forms following treatment and then validated with their temporal dynamics

**Fig. 2:**
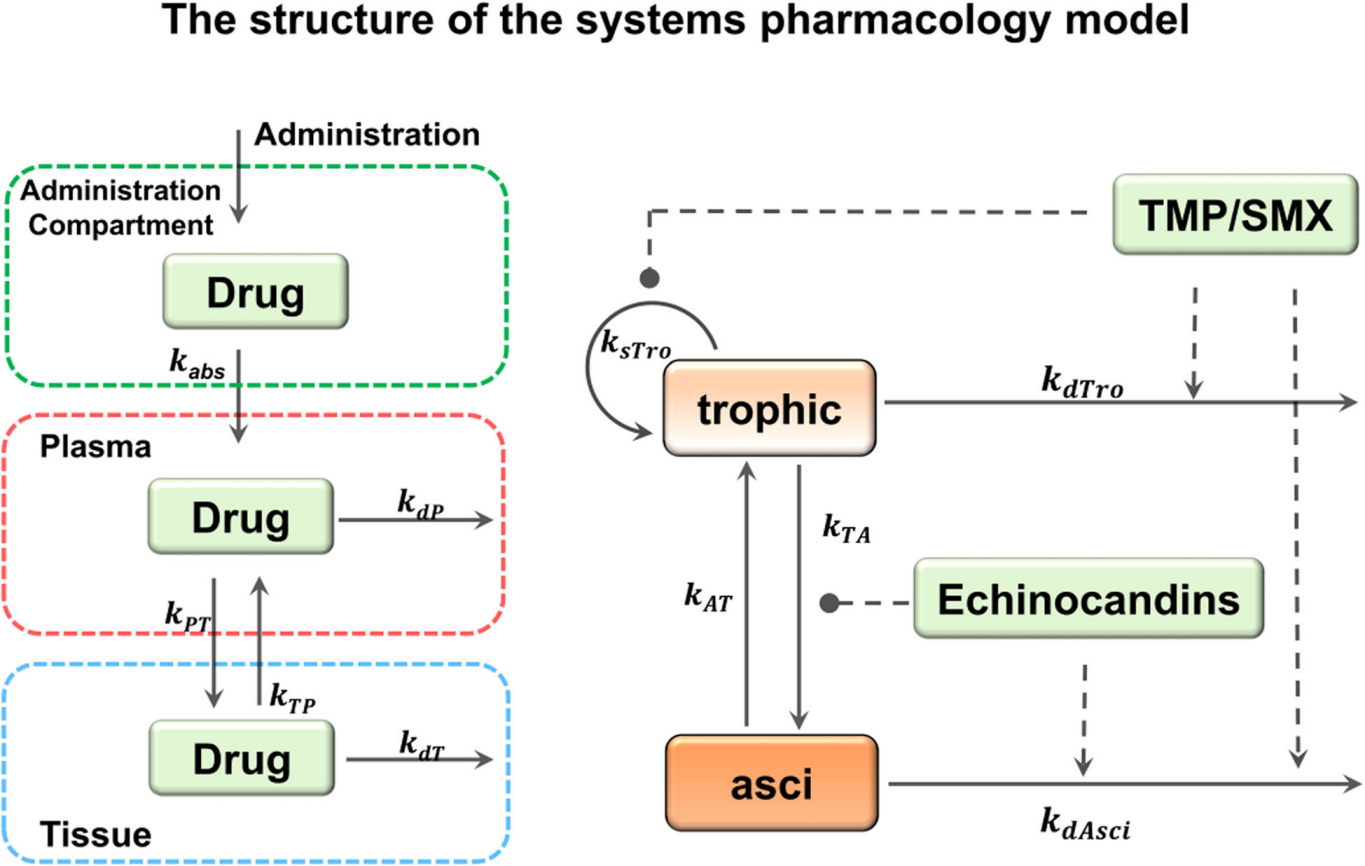
The structure of the QSP model. Left panel: A three-compartment PK module was used to describe the reported pharmacokinetic data. The first compartment was the AC, the second compartment was plasma, and the third was ‘peripheral tissue’. Drug decay was assumed to occur in plasma and ‘peripheral tissue’ compartments. The rates of drug distribution and decay were described by the corresponding parameters. Right panel: The dynamics of *Pneumocystis* were described by a two-stage model which involves both trophic forms and asci. The temporal changes of trophic forms and asci were also controlled by the indicated parameters. The drug effects were indicated by arrows (promoting) and 489 lines with solid circle heads (inhibiting)

**Fig. 3:**
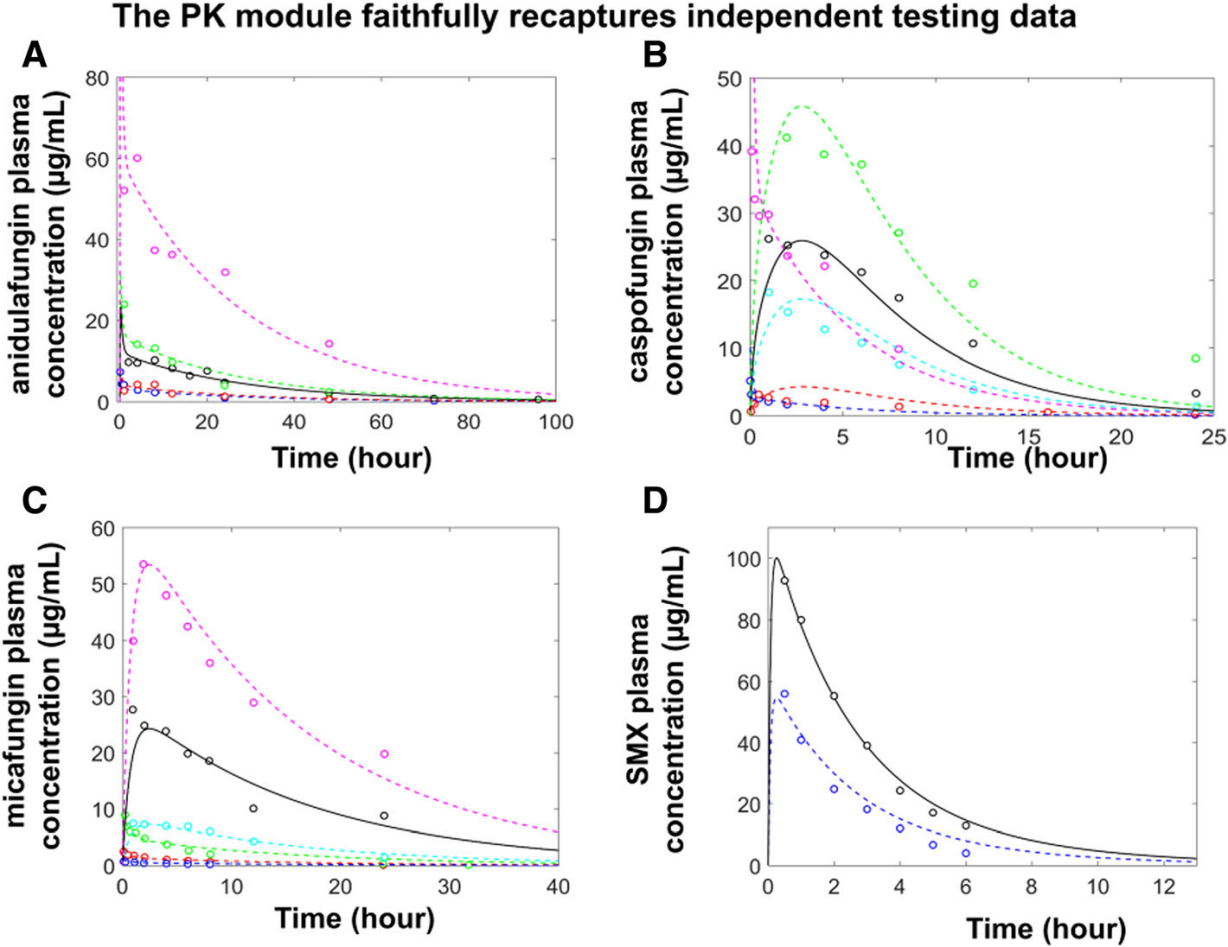
The temporal simulations of the PK modules were consistent with diverse experimental data. The temporal simulations of the plasma concentrations of anidulafungin (**a**), caspofungin (**b**), micafungin (**c**) and SMX (**d**) were compared to relevant experimental data. The black dots and black solid curves represent the construction data and corresponding model simulations; the colored dots and colored dashed curves represent the validation data and corresponding simulations. The data sources were elaborated in Table 2a. The colors in each panel were used to indicate different administration methods and dosages. In **a**, blue, *i.v*. of 1 mg/kg; magenta, green and red, *i.p.* of 80 mg/kg, 20 mg/kg and 5 mg/kg respectively. In **b**, blue and magenta, *i.v*. of 0.5 mg/kg and 5 mg; red, cyan and green, *i.p.* of 1 mg/kg, 5 mg/kg and 80 mg/kg; In **c**, blue, red and green, *i.v*. of 0.32 mg/kg, 1 mg/kg and 3.2 mg/kg; cyan and magenta, *i.p.* of 5 mg/kg and 80 mg/kg; In **d**, blue, oral of 50 mg/kg

**Fig. 4:**
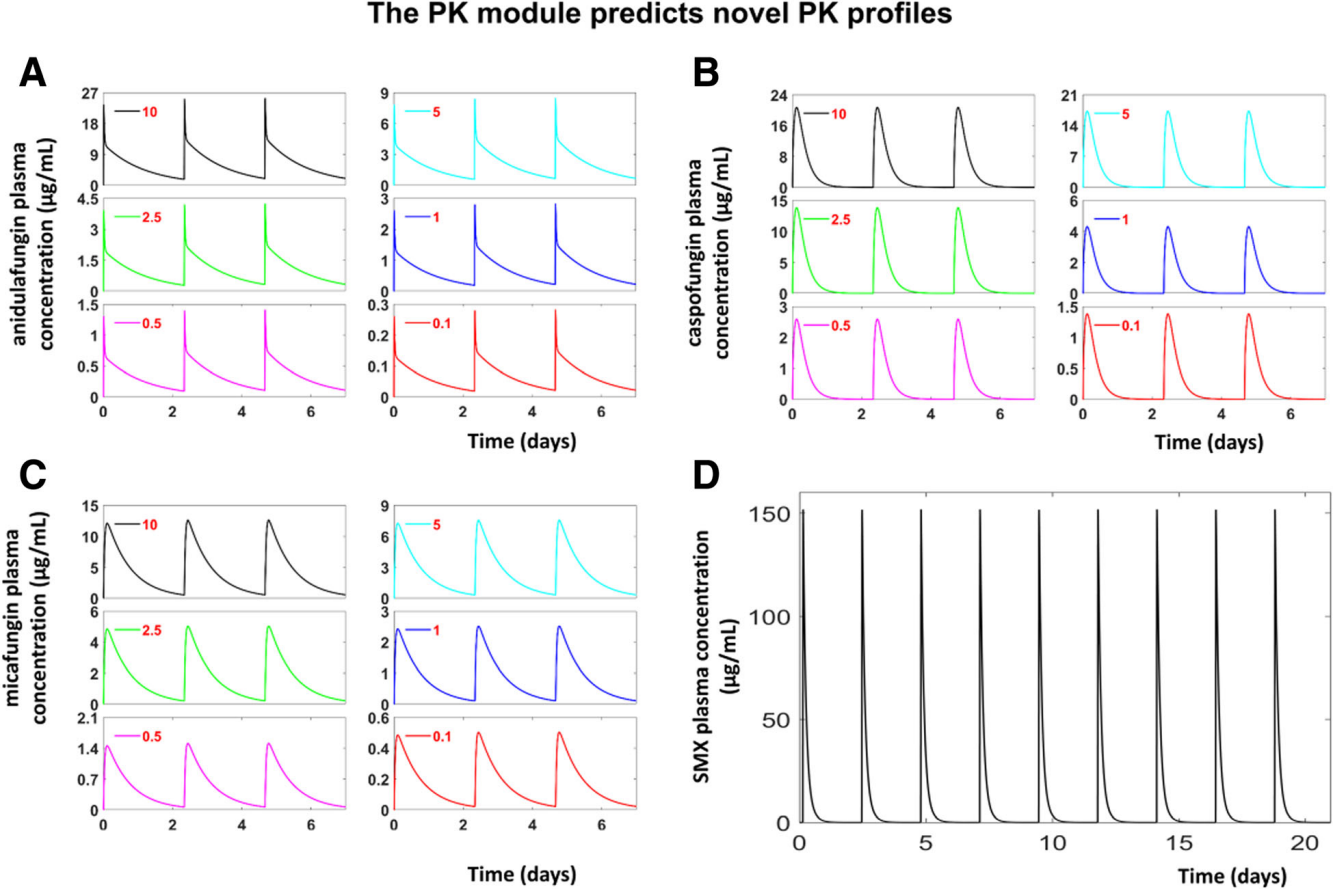
The temporal drug profiles predicted by the PK modules. **a**, **b**, **c** and **d** show the predicted plasma levels of anidulafungin, caspofungin, micafungin and SMX when administrated 3 times/week. The different dosages of anidulafungin, caspofungin, micafungin (in mg/kg) are labelled in each panel, the SMX dosage is 200 mg/kg

**Fig. 5:**
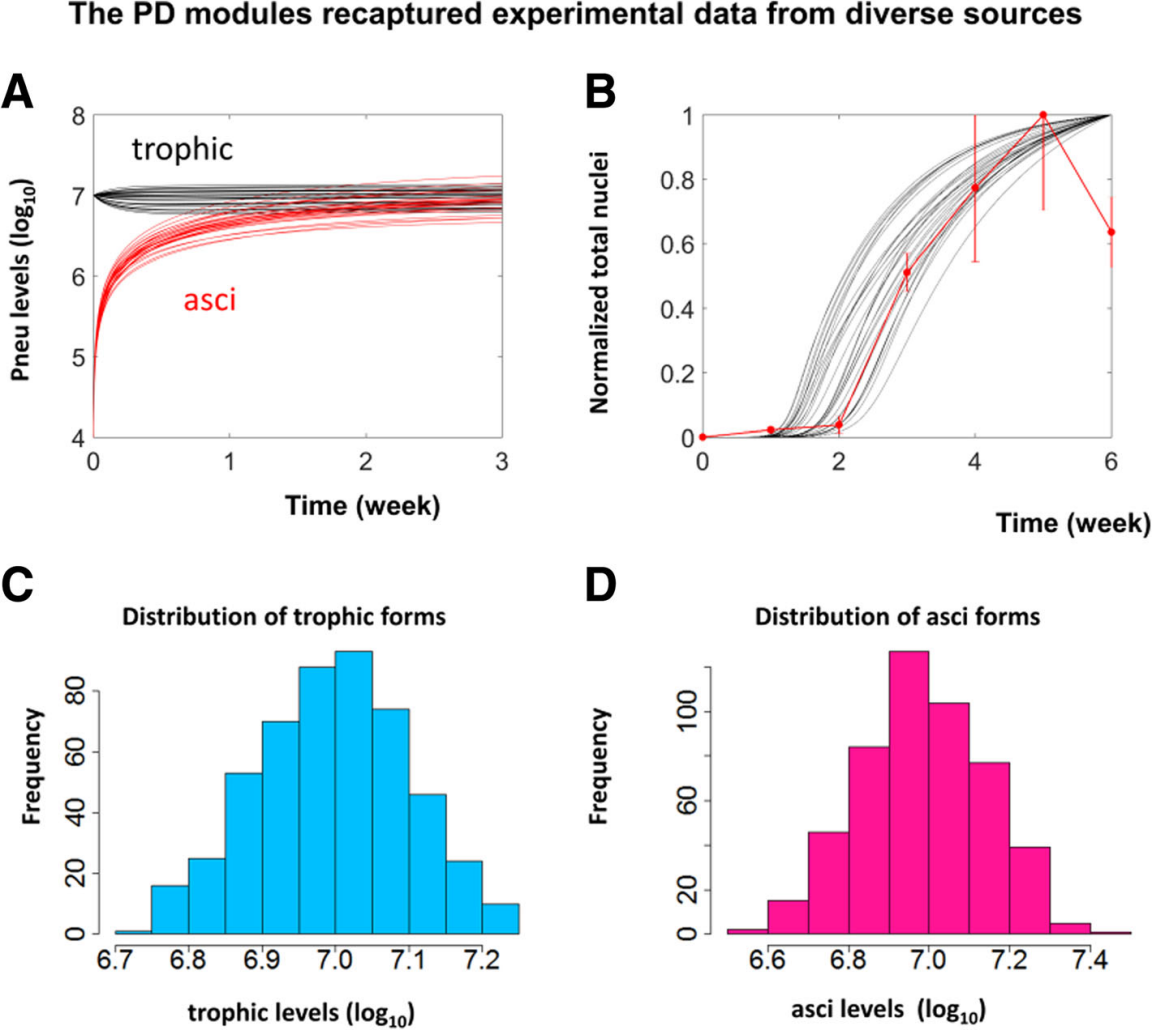
The PD modules were consistent with experimental data from diverse sources. **a** Temporal simulations for the dynamic changes of trophic form (black curves) and asci (red curves) starting from an initial state with a high level of trophic forms and a low level of asci. **b** Temporal simulations (black curves) of the normalized total number of *Pneumocystis* were compared to the normalized nuclei count from *Pneumocystis* infected mice (red dots, error bars represent SEM, n=2 or 3 for each time point). **c** and **d** Histograms showing the distributions of the numbers of the trophic form and asci simulated by the PD module

**Fig. 6:**
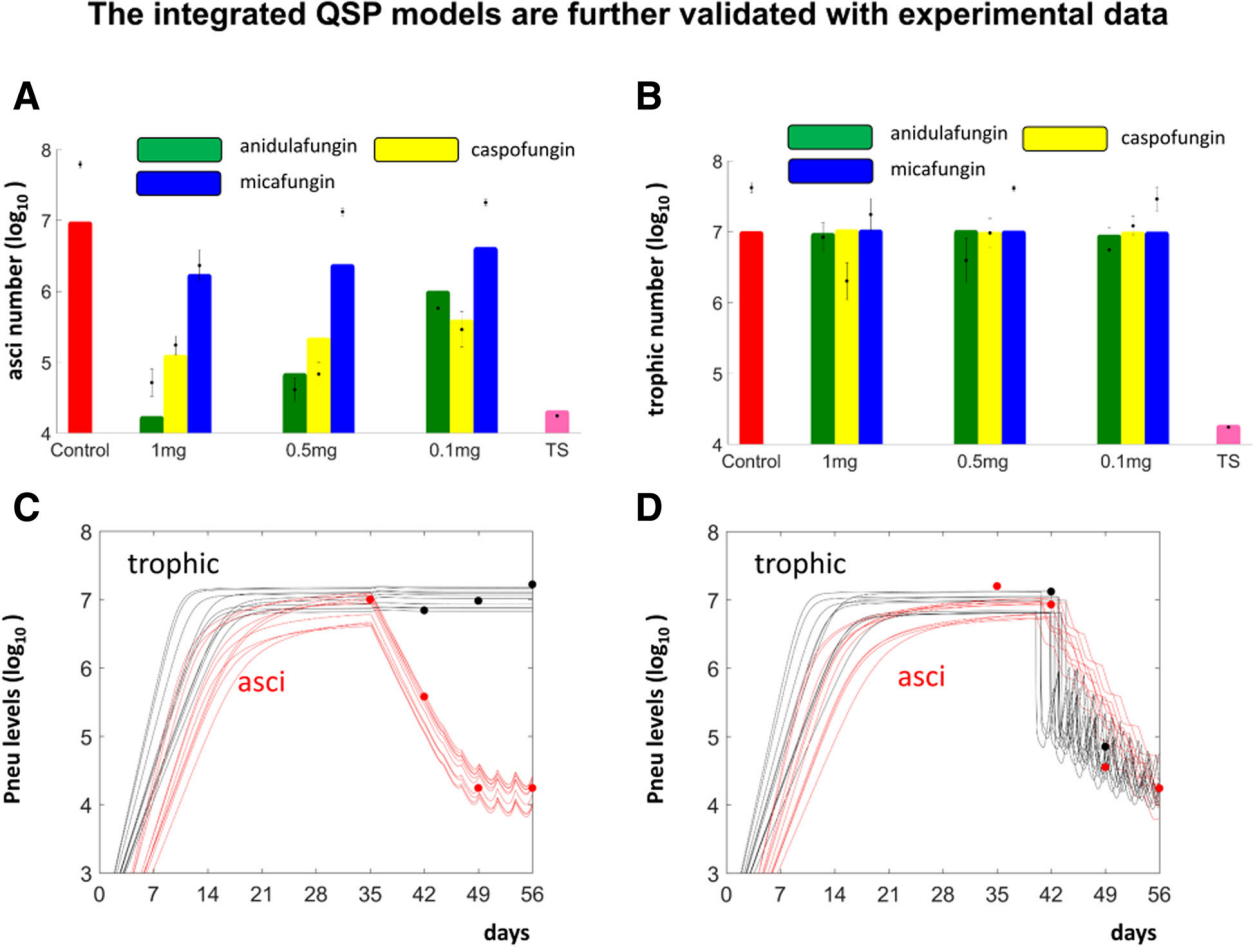
The simulations of the QSP models were consistent to relevant data. **a** and **b**. Bar plots of average simulated log10 levels: of asci (**a**) and trophic forms (**b**) at day 56 post-treatment of *Pneumocystis* from: untreated mice (Control), mice treated with varying doses of anidulafungin, caspofungin and micafungin; as well as mice treated with TMP-SMX. Corresponding experimental data are represented as dot plots with standard error. **c** The simulated dynamic changes of the trophic forms (black curves) and asci (red curves), on a log10 scale were consistent to the corresponding experimental data (black and red dots) following anidulafungin treatment. **d** The simulated dynamic changes of trophic forms (black curves) and asci (red curves) were consistent to the corresponding data (black dots and red dots) following TMP-SMX treatment

**Table 1:** There are some incorrect references to the figures in the text citations. We refer readers to the following table for correct citations

Headings in the Results	Original Figure Citation	Correct Figure Citation
The constructed PK module was validated against independent data	Figure 5	Figure 3
The PK modules predict novel PK profiles	Figure 6	Figure 4
The constructed PD modules were consistent with multiple experimental observation	Figure 3	Figure 5
Quantitative systems pharmacology model construction and validation	Figure 4	Figure 6
